# How Can Vaccines Contribute to Solving the Antimicrobial Resistance Problem?

**DOI:** 10.1128/mBio.00428-16

**Published:** 2016-06-07

**Authors:** Marc Lipsitch, George R. Siber

**Affiliations:** a Center for Communicable Disease Dynamics, Department of Epidemiology and Department of Immunology and Infectious Diseases, Harvard T.H. Chan School of Public Health, Boston, Massachusetts, USA; b ClearPath Vaccines, Rockville, Maryland, USA

## Abstract

There is a growing appreciation for the role of vaccines in confronting the problem of antimicrobial resistance (AMR). Vaccines can reduce the prevalence of resistance by reducing the need for antimicrobial use and can reduce its impact by reducing the total number of cases. By reducing the number of pathogens that may be responsible for a particular clinical syndrome, vaccines can permit the use of narrower-spectrum antibiotics for empirical therapy. These effects may be amplified by herd immunity, extending protection to unvaccinated persons in the population. Because much selection for resistance is due to selection on bystander members of the normal flora, vaccination can reduce pressure for resistance even in pathogens not included in the vaccine. Some vaccines have had disproportionate effects on drug-resistant lineages within the target species, a benefit that could be more deliberately exploited in vaccine design. We describe the effects of current vaccines in controlling AMR, survey some vaccines in development with the potential to do so further, and discuss strategies to amplify these benefits. We conclude with a discussion of research and policy priorities to more fully enlist vaccines in the battle against AMR.

## Minireview

Recent analyses of antimicrobial resistance (AMR) have focused attention on its adverse economic and health impacts and the likely growth of such harm over time (1, 2). These analyses have been accompanied by action plans to address the problem globally and nationally (3–5). These action plans focus on offering incentives to the public and private sectors to develop new antimicrobial agents and diagnostic tests and to take common sense measures such as improved infection control, antibiotic stewardship, and minimizing antibiotic use in livestock production to reduce the emergence of AMR. There is also now a growing appreciation of vaccines as a part of the solution to AMR (6–9). This minireview describes the significant contributions of current vaccines and the potential of future vaccines in controlling AMR and elucidates the mechanisms by which this can occur. It proposes several areas where further research could better quantify the impact of vaccines.

## MECHANISMS BY WHICH EXISTING VACCINES CAN ADDRESS THE AMR PROBLEM

**FIG 1:**
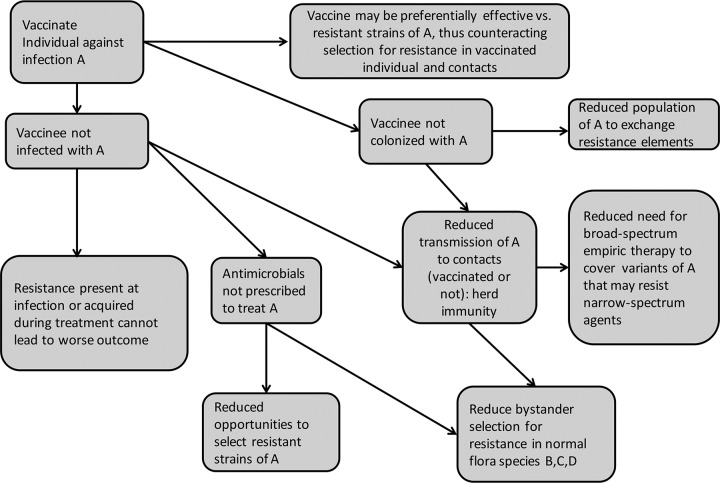
Mechanisms by which vaccines can contribute to reducing the prevalence and impact of antimicrobial resistance.

Existing vaccines already help to reduce the burden of antimicrobial resistance. Notably, resistance is not a significant clinical problem for either of the transmissible bacterial infections against which we have routinely vaccinated for decades—diphtheria and pertussis, most likely because they are rarely seen and thus rarely treated. Resistance was already becoming a problem in *Haemophilus influenzae*, *Streptococcus pneumoniae* (pneumococcus), and *Neisseria meningitidis* (meningococcus) by the time vaccines against these organisms were introduced, but the vaccines have reduced or nearly eliminated the problem. Figure 1 shows several pathways by which this may occur. Any resistant infection prevented by vaccination is a case for which, by definition, the burden of AMR disease is reduced, the need for antibiotic therapy is eliminated, and the risk of poor outcomes is avoided. Avoiding antibiotics reduces opportunities to select resistant variants of the targeted pathogen, and of other, “bystander” species that are susceptible to the antibiotic (10). In some cases, the elimination of a specific pathogen by vaccination reduces the need to use broad-spectrum antibiotics for empirical treatment of a clinical syndrome, such as pneumonia, by eliminating the need to “cover” possibly resistant pathogens that are no longer likely to be the causes of that syndrome.

The benefits of vaccines in combating AMR by each of these mechanisms can be amplified by the indirect protection, or herd immunity (11), that results when vaccinated individuals do not themselves become infected or colonized, and hence do not transmit the pathogen to others. In this way, infections, resistant infections, and antimicrobial use can be reduced not only in vaccinated individuals but also in their contacts.

Finally, for vaccines against organisms like *S. pneumoniae*, *Staphylococcus aureus*, and members of the family *Enterobacteriaceae*, which asymptomatically colonize the nasopharynx, skin, gut, or other sites, there is the theoretical possibility that reducing the density of microbial populations by vaccination reduces the opportunities for genetic exchange of resistance elements (12, 13).

Each of these effects, apart from the last, has been documented for one or more existing vaccines, though our level of certainty about the magnitude of each effect varies by vaccine and population. Some prominent examples are given in the following subsections.

### Hib conjugate vaccine.

The introduction of *Haemophilus influenzae* type b (Hib) conjugate vaccine has virtually eliminated Hib meningitis, bacteremia, pneumonia, and epiglottitis in regions where it has been widely deployed, including drug-resistant infection (14). By 1990, when this vaccine was licensed for infants, Hib had already evolved resistance to ampicillin, driving recommendations to use chloramphenicol and broad-spectrum cephalosporins for empirical treatment of meningitis. The elimination of this clinical problem by vaccination, including a major impact on unvaccinated persons through herd immunity (15), reduced the need for antibiotics and preempted the continuing evolution of multiple resistance and the narrowing of therapeutic options that would likely have ensued had Hib disease remained a threat. The continued evolution of resistance without vaccination is illustrated by limited data from India (16), where introduction of Hib vaccine was delayed.

### PCV.

The pneumococcus is another example of a pathogen for which vaccination reduced drug-resistant disease, primarily through reducing the overall burden of disease but also by targeting the most resistant serotypes. In the United States, introduction of the seven-valent pneumococcal conjugate vaccine (PCV7), which included five serotypes that accounted for 78% of penicillin nonsusceptibility, was associated between 2000 and 2004 with a 57% reduction in the incidence of penicillin-nonsusceptible invasive pneumococcal disease (IPD) and an 84% decrease in the rate of multidrug-resistant IPD (17). Other countries also saw declines in resistant IPD following vaccine introduction (18). Due to the decreased need for treating IPD and severe otitis media (19), the use of antibiotics has demonstrably declined in young children (20).

In contrast to the Hib example (21), however, universal use of PCV-7 led to increased disease from certain nonvaccine serotypes (22, 23), particularly type 19A, which also had high rates of penicillin nonsusceptibility and eroded the gains against resistant disease. Introduction of 13-valent PCV in 2010, which contains 6 additional types, including 19A, further reduced the incidence of IPD and of antibiotic-resistant pneumococci (24).

Extending the use of Hib and PCV could further dramatically reduce antibiotic use. It has been estimated that introduction of Hib conjugate and PCV-13 to 75 developing world countries could reduce antibiotic use for these diseases by 47% and avert 11.4 million days of antibiotic use in children younger than <5 years old each year (6). A vaccine’s impact on antimicrobial use may be disproportionate to its impact on severe disease, because at least in the United States, mild infections such as otitis media are the most frequent indications for antimicrobial use (25).

The benefits of such vaccines for resistance may be greatest when they are first introduced. Interestingly, the proportion of individuals colonized by pneumococci is essentially unchanged after introduction of PCV-7 and PCV-13, although the incidence of invasive disease declined with the near-disappearance of vaccine serotype pneumococci. This leaves nonvaccine serotypes (NVTs) in the nasopharynx where they are subject to pressure to evolve AMR, as they have begun to do (26). If it were possible to reduce the density and prevalence of colonization by all pneumococcal serotypes, a potential benefit is that the ability of the organism to exchange genetic information (which occurs primarily in the human nasopharynx by an efficient process of transformation) and evolve resistance to antimicrobials or vaccines would be severely curtailed. Vaccines utilizing whole pneumococcal cells or conserved proteins that induce Th17 type T cell responses have been shown to prevent or reduce pneumococcal colonization in animals and are currently being evaluated in humans (27).

### Respiratory virus vaccines.

Vaccines against influenza virus reduce the incidence of influenza, which infects a significant proportion of the population each year and causes ca. 200,000 hospitalizations in the United States. By preventing a proportion of these cases, vaccines reduce both appropriate and inappropriate antimicrobial prescribing. In the United States, a recent estimate is that one third of antibiotic prescriptions in ambulatory care are inappropriate, with a large proportion of inappropriate prescribing attributable to acute respiratory infections (28). Many of these inappropriately-treated infections may have been caused by influenza (29, 30) or other viruses and could be prevented by vaccinating against such viruses. Moreover, influenza vaccination can also prevent cases of influenza that would have led to secondary bacterial infections that would have prompted appropriate antibiotic treatment.

The temporal correlation between influenza incidence and some types of antimicrobial use is striking (31), suggesting that vaccine-induced reductions in influenza could lead to reductions in selection pressure caused by antibiotic treatment of influenza symptoms (30). A Canadian ecological study estimated that antibiotic prescriptions during the influenza season were reduced more that 60% after introduction of a universal seasonal influenza immunization program (32).

A vaccine against respiratory syncytial virus (RSV), such as the vaccine currently in phase 3 trials (33), deployed against infections in mothers and children and in the elderly could have a similar beneficial impact, as could other, future respiratory viral vaccines.

## PROSPECTS FOR GAINING SIMILAR BENEFITS WITH NEW OR IMPROVED VACCINES AGAINST OTHER AMR PATHOGENS

Vaccines of particular interest are those targeting the most important causes of health care-associated infections (HAI) which are frequently resistant to multiple antibiotics (1, 10). The most common causes of HAI include multiply resistant Gram-negative bacteria; recent publications report isolates from across the globe that have become resistant to the last-resort agents, polymyxin and colistin (34, 35). Resistance to first- and second-line agents is also a problem in Gram-positive organisms such as *Staphylococcus aureus* and enterococci, and infection with *Clostridium difficile* is an important complication of antimicrobial therapy. *Candida* species are important causes of mucosal and disseminated infections in immunocompromised patients and as a consequence of antimicrobial therapy (1).

With improvements in vaccine technologies and improved understanding of immunologic defenses, the development of vaccines against these pathogens is now feasible and has a strong likelihood of success. Many of these pathogens have surface polysaccharides for which vaccines are highly protective in animal models especially when linked to carrier proteins in the form of conjugates. This is the technology that has been used for the highly successful vaccines against Hib, pneumococci and meningococci. The wide diversity of these polysaccharides will pose a significant challenge, as was the case for pneumococci. However, as for pneumococci, not all of the numerous O and K polysaccharides of the Gram-negative bacteria are associated with clinical disease, particularly invasive disease. Consequently, it may be possible to target a smaller number of serotypes of *Escherichia coli*, *Klebsiella* spp., and *Pseudomonas aeruginosa* that are responsible for the majority of resistant HAI (36, 37). In addition, new technologies have become available that simplify the manufacture of polyvalent polysaccharide conjugate vaccines by synthesis within genetically engineered *E. coli* (38) or by simple high-yield complexing of biotinylated polysaccharides with carrier protein-avidin fusions (39).

Vaccination targeting virulence determinants may also be an effective approach for HAI pathogens, enhanced by the current availability of multiple genome sequences for most species of interest and technologies such as reverse vaccinology to screen potential candidates for immunogenicity, protection in animal models, and a role in virulence (40). Virulence factors such as toxins and adhesins are widely conserved among pathogenic members of a species such as *E. coli* but are not found in the commensal members (41, 42). These conserved virulence factors are potentially ideal antigens of multicomponent vaccines directed at many of the HAI pathogens since they would have the advantage of selectively eliminating the pathogens and leaving the commensal organisms undisturbed. Similar approaches may be useful for other pathogens such as *Clostridium difficile*, which has pathogenic and commensal members (43).

Examples of vaccines against HAI that are currently in clinical development and that are using the principle of selectively targeting virulence factors include the following: (i) a four-component vaccine containing two capsular polysaccharides and two virulence-associated proteins (ClfA and MntC) against *S. aureus* which is currently in phase 2b trials (44), (ii) three vaccines against *Clostridium difficile* based on toxins A and B which are in phase 2 and 3 trials (45), (iii) a vaccine against *Pseudomonas aeruginosa* based on conserved outer membrane protein F/I fusion which is in phase 2/3 trials in ventilated intensive care unit (ICU) patients (46), and (iv) a vaccine against *Candida* based on a T cell target protein, Als3 (47, 48), which is in phase 2 trials. *Staphylococcus aureus* is a particularly difficult target because of its multiple and apparently redundant virulence factors (49) and the absence of good animal models. This has led to multiple vaccine failures (50, 51).

There are also a number of therapeutic monoclonal antibodies in development for HAI infections which are designed for therapy together with antibiotics or for prophylaxis in very high-risk patients such as those on mechanical ventilation (reviewed in reference 52). The targets of these antibodies include the toxins of *C. difficile*, leukotoxins and cytotoxins *of Staphylococcus aureus*, and the O polysaccharide, the PsI exopolysaccharide, or the type 3 secretion pathway (PcrV) of *Pseudomonas aeruginosa* (52). If these targets can be validated in therapy, they will become important components of active vaccines. Ideally, vaccines can be developed that not only provide systemic protection but also reduce colonization by the pathogen with the consequence that the numbers of organisms subject to selective pressure and transmission of resistant organisms would be reduced.

Achieving widespread protection and even herd immunity against HAI pathogens might be challenging for several reasons, including environmental reservoirs for some of them, the practical challenges of vaccinating a large proportion of the population against pathogens that are largely restricted to hospitals, and the possibility that vaccines might not strongly protect against colonization. On the other hand, for several of the directly transmitted infections without an important environmental reservoir, it is possible that the design of vaccines to induce helper T cell responses will provide a new way to reduce colonization in humans. It has been suggested that the whole-cell pertussis vaccine induced Th1 and Th17 responses and protected against colonization and transmission of *Bordetella pertussis*, whereas the acellular vaccine induces mainly Th2 responses which do not affect colonization (53, 54). This may indicate that it is indeed feasible to induce T cell-mediated immunity against mucosal colonization with an appropriately designed vaccine.

Other infections for which drug resistance is currently a problem and for which new vaccines (or improved vaccines) appear to be within reach include malaria (55), tuberculosis, nontyphoidal *Salmonella*, *Shigella*, and respiratory infections with nontypeable *H. influenzae* (56). Vaccines against *Neisseria gonorrhoeae* that were under active development some years ago need to be revived, since this organism is showing increasing resistance to the last major classes of appropriate antimicrobial agents, macrolides and cephalosporins (57).

## TARGETING VACCINES SELECTIVELY TO RESISTANT CLONES OR DIRECTLY AGAINST FACTORS MEDIATING RESISTANCE: A NOVEL APPROACH TO CONTROLLING AMR

Completely protecting a vaccinated individual against disease and (if applicable) mucosal colonization with all strains of a pathogen is almost certainly the best way to achieve a reduction in disease burden, a reduction of selection pressure from antimicrobial treatment directed at that pathogen, and a reduction of the pool of organisms that can exchange resistance genes. However, it is not the only vaccine strategy that can aid in countering AMR.

For many mucosal colonizing bacteria, vaccines have so far been unable to prevent colonization altogether. For some opportunistic pathogens that are members of our normal flora, such as *E. coli*, it may not even be desirable to eradicate the entire species with vaccines, even if it were possible. In this section, we suggest a strategy that turns this limitation into a tool to counteract selection for resistance.

It has recently been proposed that targeting vaccines against resistant strains or even against resistance determinants themselves may be an effective way to counteract selection pressure for antimicrobial resistance (58, 59). The selection pressure imposed by antimicrobial use is intense but localized: individual patients are treated, exerting very strong selection on their pathogen populations, but only on their populations. Vaccines, too, exert selection pressure, against the strains in the vaccine and, sometimes, in favor of strains that can escape from vaccine-induced immunity or are not targeted by the vaccine. Such selection has been observed clearly in *S. pneumoniae* with the phenomenon of serotype replacement (60) and has been considered a possibility in the case of hepatitis B (61), meningococcus (62), and pertussis (63, 64).

### Selectively targeting resistant clones.

Antibiotic resistance is frequently maintained and spread by particularly successful clonal strains of a pathogen. Spread may be mediated by a wide variety of virulence factors such as toxins, adherence factors, or factors that enable the organism to evade host defenses. In principle, vaccines against such virulence factors are a valid approach to target AMR infections as discussed above.

As previously mentioned, the seven-valent pneumococcal conjugate vaccine targeted the five serotypes that had the highest level of penicillin nonsusceptibility and thus substantially reduced AMR in this pathogen. This led to a decline in resistance, eroded over time by the increase in resistant nonvaccine types, and repeated with the introduction of PCV13, which contained the most resistant of the common serotypes in disease, serotype 19A (24).

Many toxins are clonally associated with methicillin resistance in *S. aureus* (65), including by close genetic linkage (66). Vaccines against a resistance-associated toxin have been effective in an animal model (67). By analogy to the pneumococcal experience, vaccines targeting these toxins might disproportionately reduce the frequency of resistant or even multiply resistant (10) strains. A limitation of any strategy targeting antigens that are associated with resistance determinants, however, is that recombination may erode that association over time, reducing the disproportionate effect on resistance (68), as appears to have happened with the seven-valent pneumococcal conjugate vaccine (26, 69).

### Vaccines directly targeting resistance determinants.

The strategy of targeting resistance determinants themselves has the appeal that it would exert consistent selection against resistance, if effective immune responses could be generated. It has the disadvantage that these targets are limited in number, may not be very immunogenic, or may fail to induce protective immune responses.

A few promising animal studies of vaccines directly targeting resistance determinants have been published. Two of these indicate that resistance determinants can be the basis of vaccines that are immunogenic and protective against methicillin-resistant *S*. *aureus* (MRSA) (70), where the target is the resistance-conferring extra penicillin-binding protein (PBP2a) and in *Neisseria meningitidis*, where the target was one of the core penicillin-binding proteins; activity against different alleles was demonstrated (71). Another study showed enhancement of ceftazidime treatment of *Pseudomonas aeruginosa*, in those animals that produced strong neutralizing antibody responses to immunization with AmpC beta-lactamase protein (72).

These considerations help define types of pathogens for which antiresistance vaccines might be most likely to be effective.

First, one could target resistance determinants that are immunogenic and for which responses are effective at the site of transmission, typically the mucosal surface. The major benefit of counterselecting resistance with a vaccine would be at the population level, not within an individual host. Thus, eliciting immune responses effective at the site of transmission (e.g., nasopharynx, gastrointestinal [GI] tract) would be more important than effectiveness at the site of pathogenesis (e.g., bloodstream, urinary tract). It follows that such vaccines would be maximally effective only in mass immunization programs. This might include vaccination of agricultural animals to reduce resistance in foodborne human pathogens.

Second, antiresistance vaccines should be more effective against drug-resistant strains than against drug-susceptible strains, either by specifically targeting resistant alleles of a conserved protein (such as a penicillin-binding protein in bacteria or neuraminidase in influenza virus) or by targeting proteins uniquely present in resistant isolates (such as beta-lactamases or ribosomal methylases conferring macrolide resistance). This additional effectiveness may be modest, as small as a few percent (58, 59), because the large number of vaccinated hosts amplifies the modest selective effect to counteract the stronger, more-concentrated selective effect of antimicrobial treatment.

Third, given that persistence of competing bacteria within the colonizing site can therefore be an advantage for antiresistance vaccines, their use may be particularly promising in the context of multiantigen vaccines or as carrier proteins for glycoconjugate vaccines, as these typically do not achieve full sterilization of the colonizing population and already include multiple antigens. One could consider modifying existing or candidate (73) glycoconjugate vaccines to use resistance determinants as the protein carrier or adding a resistance determinant as an additional component to a multicomponent vaccine.

## SYNERGY BETWEEN PASSIVE OR VACCINE-INDUCED ANTIBODIES AND ANTIMICROBIALS IN TREATING OR PREVENTING AMR INFECTIONS

A number of studies have evaluated the potential for polyclonal or monoclonal antibodies to act synergistically with antibiotics in treating infections (52). Vaccines that actively induce such antibodies to appropriate bacterial antigens would be expected to have similar benefits.

*In vitro* studies have evaluated the synergistic effects of antibodies to efflux pumps with antibiotics. A polyclonal antibody to an ATP-binding cassette efflux pump of *Stenotrophomonas maltophilia* had synergistic or additive effects with a variety of antibiotics, including co-trimoxazole, ticarcillin-clavulanate, and ciprofloxacin against this highly resistant HAI pathogen (74). Another polyclonal antibody against the FloR efflux pump inhibited antibiotic accumulation of the chloramphenicol analogue, florfenicol in *E. coli* (75). A bifunctional antibody to *P. aeruginosa* directed against both the exopolysaccharide Psl and the type III secretion system virulence factor PcrV synergized with multiple classes of antibiotics and even against drug-resistant strains (76). Similar examples of antibodies enhancing antibiotic action have been shown for monoclonal antibodies to *P. aeruginosa* O11 lipopolysaccharide and meropenem in a lung infection model (77), for antistaphylococcal alpha-toxin and linezolid or vancomycin in a mouse pneumonia model (78), with monoclonal antibody to anthrax protective antigen and ciprofloxacin in a rabbit inhalational anthrax model (79), and with monoclonal antibody to *Candida* heat shock protein 90 and amphotericin B in murine systemic candidiasis (80).

## RESEARCH AND POLICY NEEDS

To make appropriate investments in research and development of vaccines as part of the response to AMR, it will be necessary to quantify as well as possible the likely impact of existing vaccines and of candidate vaccines by each of the mechanisms described here.

A first step is to quantify the proportion of resistant disease that is likely to be affected by the use of a vaccine. If the vaccine is equally effective against all strains of a pathogen, then its initial effect on resistant disease incidence will be pro rata and easily estimated. If as in the case of pneumococcal conjugate vaccines, activity is strain specific, estimates of the prevalence of resistance in vaccine-targeted strains relative to the whole population will provide a starting point for estimating the reduction in the incidence of resistant disease anticipated from vaccine use.

More-detailed work will be required to estimate the impact of vaccines on reducing selection for resistance. In clinical trials, all-cause and cause-specific antimicrobial prescriptions can be an informative endpoint to quantify reductions in prescribing. Such declines in antimicrobial use might be reduced, not only by vaccines targeting an antimicrobial-treatable organism (such as the pneumococcus) but also by vaccines against pathogens that produce symptoms that are often inappropriately treated with antimicrobials (such as influenza virus and respiratory syncytial virus). Before the clinical trial stage, observational studies of the association between vaccine-preventable diseases and antimicrobial prescribing as an outcome can attempt to estimate “attributable prescribing.” Such studies have been rare so far (31) and may be methodologically challenging. Methodology developed to estimate influenza-attributable mortality (81) could be adapted to estimate influenza-attributable antimicrobial prescribing. These studies will have to take into account the biology of the particular infection. For example, malaria vaccines may have the effect of reducing symptomatic infection more than total infection (82), thereby reducing the proportion of all cases treated with antimalarial drugs, a slightly different mechanism from those discussed so far.

For vaccines that reduce colonization of the targeted organisms, methodologies should be refined to more accurately predict the magnitude of herd immune effects, including reduced transmission of resistant organism, reduced disease, and reduced need for antibiotic treatment in the entire population. It would also be useful to model the effects of reduced colonization by one species on its capacity to evolve AMR or regain virulence and also on the potential for replacement by other pathogens occupying the same niche.

Designing a vaccine to specifically target resistance determinants or resistant lineages is in early stages, but the idea may be promising (83). The first step is clearly to explore in multiple systems whether resistance determinants, or antigens strongly associated with them, can be immunogenic and protective. For surface antigens, elicitation of antibody is the most obvious mechanism, but the growing interest in T cell-based vaccines, capable at least in theory of protective responses to both surface and nonsurface structures, expands the possibility of targeting resistance determinants that may not be surface exposed, such as Gram-negative beta-lactamases or ribosomal methylases. It would be highly relevant to determine whether such resistance factors could mediate Th1 or Th17 immunity which reduces mucosal colonization by the target pathogen. The potential for immune mechanisms such as antibodies or T cells to synergistically increase the susceptibility of highly resistant pathogens to antimicrobial agents deserves further evaluation.

Given the theoretical prediction that even very weak selective pressure exerted by a vaccine could shift the balance against resistant strains, new assays will need to be developed that are capable of detecting such weak selective pressure. *In vivo* competitive assays have been used to detect modest fitness differences between strains of the same species by comparing resistant to susceptible strains growing together in the upper respiratory tract of an infant rat (84) or mouse (85). By comparing the competitive results of resistant and susceptible strains in vaccinated versus unvaccinated animals, the ability of a vaccine to select against resistance could be evaluated. If promising candidates were identified, there would be a need to identify ways to study these vaccines clinically, not only for their antiresistance effects but also for their direct impacts on protecting patients against disease.

From a policy perspective, the appropriate recent focus on the failure of markets to ensure access, conservation, and innovation in the antimicrobial drug marketplace (86) should be broadened to include incentives for vaccines that can help meet the end goal of reducing the need for antimicrobial treatment while making sure the drugs are effective when they are needed (6–8, 86). Attention to appropriate incentives for vaccines is especially relevant in light of economic arguments that markets tend to provide weaker incentives for developing vaccines than for drug development (87).

## CONCLUSION

Vaccines and antibiotics are widely hailed as the two greatest accomplishments of modern medicine. In fact, vaccines are the medical intervention that has saved the most lives globally. As evolution begins to erode the value of antibiotics, a multipronged approach to preserving and restoring this value is needed. Vaccines have an important role to play in doing so.
